# Hemolymph microbiota and host immunity of crustaceans and mollusks

**DOI:** 10.1093/ismejo/wraf133

**Published:** 2025-06-28

**Authors:** Rohit Rathour, Yingxue Ma, Jinbo Xiong, Xian-Wei Wang, Jillian Petersen, Xinxu Zhang

**Affiliations:** Archaeal Biology Center, Synthetic Biology Research Center, Shenzhen Key Laboratory of Marine Microbiome Engineering, Key Laboratory of Marine Microbiome Engineering of Guangdong Higher Education Institutes, Institute for Advanced Study, Shenzhen University, 3883 Baishi Road, Shenzhen, Guangdong, 518060, China; College of Life Science and Oceanography, Shenzhen University, 1066 Xueyuan Blvd, Shenzhen, Guangdong, 518055, China; Archaeal Biology Center, Synthetic Biology Research Center, Shenzhen Key Laboratory of Marine Microbiome Engineering, Key Laboratory of Marine Microbiome Engineering of Guangdong Higher Education Institutes, Institute for Advanced Study, Shenzhen University, 3883 Baishi Road, Shenzhen, Guangdong, 518060, China; Key Laboratory of Aquacultural Biotechnology, Ministry of Education, School of Marine Sciences, Ningbo University, 169 Qixing South Road, Ningbo, Zhejiang, 315832, China; Shandong Provincial Key Laboratory of Animal Cells and Developmental Biology, School of Life Sciences, Shandong University, 72 Binhai Road, Qingdao, Shandong, 266237, China; Centre for Microbiology and Environmental Systems Science, University of Vienna, Djerassiplatz 1, 1030 Vienna, Austria; Doctoral School in Microbiology and Environmental Science, University of Vienna, Djerassiplatz 1, 1030 Vienna, Austria; Archaeal Biology Center, Synthetic Biology Research Center, Shenzhen Key Laboratory of Marine Microbiome Engineering, Key Laboratory of Marine Microbiome Engineering of Guangdong Higher Education Institutes, Institute for Advanced Study, Shenzhen University, 3883 Baishi Road, Shenzhen, Guangdong, 518060, China; Key Laboratory of Aquacultural Biotechnology, Ministry of Education, School of Marine Sciences, Ningbo University, 169 Qixing South Road, Ningbo, Zhejiang, 315832, China

**Keywords:** crustaceans, hemolymph microbiome, innate immunity, mollusks, multi-omics

## Abstract

Crustaceans and mollusks have major economic importance and are also key players in aquatic biogeochemical cycles. However, disease outbreaks, temperature fluctuations, pollutants, and other stressors have severely threatened their global production. Invertebrates generally rely on their innate immune system as the primary defense mechanism, operating at cellular and humoral levels to protect against pathogens. The hemolymph plays a vital role in immune responses, containing microbial communities that interact with the host’s immune processes. Significant advances in molecular methods such as metagenomics, metatranscriptomics, metaproteomics, and metabolomics have revealed the presence of a resident hemolymph microbiome and delineated its potentially vital role in immune homeostasis and overall host health. Accordingly, understanding the composition and role of the hemolymph microbiota, alongside innate immune responses, has become a key focus in recent research aimed at unraveling disease resistance mechanisms and supporting sustainable aquaculture practices. Here, we summarize the latest advancements in understanding the host and environmental factors that shape hemolymph microbiota diversity in various crustacean and mollusk species. We also consider the innate immune responses of the hosts, as these modulate interactions between hosts, microbes, and environments. Interactions within the hemolymph microbiome significantly affect host health, providing critical insights for advancing sustainable aquaculture.

## Introduction

Aquatic invertebrates such as crustaceans and mollusks are among the most species-rich groups of invertebrates and comprise some of the key farmed species in the aquaculture sector. As reported by the Food and Agriculture Organization (FOA) of the United Nations in 2024, the aquaculture sector generated USD 313 billion from aquatic animals in the year 2022, with crustaceans contributing 24.6% and mollusks another 15.6% [1]. In addition to their economic importance, these invertebrates are also vital to aquatic ecosystems, aiding nutrient cycling and energy transfer in food webs and acting as indicator species and filter feeders [2, 3]. Like most other animals, invertebrates such as crustaceans and mollusks host specific communities of microorganisms that influence their health, development, ecology, and evolution [4–6]. Changes in aquatic ecosystems due to anthropogenic activities and other natural factors significantly impact relationships between aquatic animals and their associated microbial communities [7]. Understanding these interactions is crucial for maintaining health and homeostasis in a changing world, as they influence host growth, development, and resistance to biotic and abiotic stressors [8, 9].

Previously, microbiome research in aquatic invertebrates was mainly focused on gut microbiomes. These studies have improved our understanding of microbial community responses to environmental perturbations and host-associated factors such as diet, age, sex, disease status, drug exposure, and nutrient absorption [10–13]. Furthermore, they revealed compositional and functional shifts related to the presence of, and response to, a range of pathogenic infections including viral (e.g. white spot syndrome virus [WSSV], decapod iridescent virus [DIV], infectious myonecrosis virus [IMNV]), bacterial (e.g. *Aeromonas hydrophila*, *Vibrio* spp.), fungal (e.g. *Aspergillus* spp., *Candida* spp., *Fusarium* spp.), and various others, which vary across different host species [12, 14–18]. In recent years, there has been increased interest in identifying how invertebrate-associated microbes interact with the host’s immune system [19]. In aquatic invertebrates, particularly bivalves and crustaceans, hemolymph serves as the primary circulatory fluid, functionally analogous to blood in vertebrates. It interacts directly with the animal’s tissues, regulating innate immune responses [20]. However, not all invertebrates possess hemolymph or share similar circulatory architectures, as seen in groups such as sponges, corals, and cephalopods [21]. Primarily composed of liquid plasma and immune cells called hemocytes, the hemolymph is considered a nutrient-rich fluid due to its balanced ions and near-neutral pH, as well as its essential carbohydrates, amino acids, peptides, and proteins, which would provide an ideal environment for microbial life [22–24]. It was previously thought to be free of microbes, but research from the last decade is beginning to reveal that the hemolymph of various healthy aquatic invertebrates may have a resident microbial community [25, 26]. Semi-open circulatory systems (crustaceans) and open circulatory systems (most mollusks) allow hemolymph to flow through open sinuses rather than being entirely confined within blood vessels. This anatomical feature increases the exposure of hemolymph to both internal tissues and environmental microbes, thereby providing a pathway for microbial exchange and colonization. Accordingly, determining microbial communities in other internal compartments, such as hepatopancreas and gills, could complement hemolymph studies and provide a broader view of host–microbe interactions within semi-open systems [27]. Several methodological advances, including microscopy and high-throughput sequencing technologies (including amplicon sequencing (16S/18S ribosomal RNA (rRNA) genes/internal transcribed spacer region), shotgun metagenomics, metatranscriptomics, metaproteomics, and metabolomics) have revolutionized the study of the hemolymph microbiome in aquatic invertebrates, contributing to a better understanding of microbial abundance and diversity in this habitat [26, 28–31]. Numerous studies have now focused on exploring the diversity, stability, and functional roles of hemolymph microbial communities alongside the factors that shape their influence on host homeostasis [19, 32]. This review focuses on the hemolymph microbiome of aquatic crustaceans and mollusks. For information on the hemolymph microbiome of terrestrial insects, we direct the reader to the outstanding review of Blow and Douglas [24].

Various environmental and biological factors shape the diversity and abundance of hemolymph microbiota in aquatic invertebrates, reflecting the complex interplay between external ecosystems and internal host processes. These factors determine the hemolymph microbiota composition and mediate interactions between the host and its microbiome, which can profoundly influence physiological processes, immune function, and overall health. In light of this, and considering the increasing anthropogenic pressures on aquatic ecosystems, including climate change, pollution, and habitat alteration, it is essential to understand how these environmental disturbances influence hemolymph-associated microbial communities. Incorporating such perspectives is critical for ecological risk assessment and for developing microbiome-informed indicators of environmental stress in aquaculture and natural settings. Given the emerging recognition of the important role of hemolymph microbiota in aquatic invertebrates, this review summarizes the various factors known to influence microbiome composition within the hemolymph. We begin by examining environmental factors and host-specific determinants that affect hemolymph microbiota. We explore the dynamic symbiotic interactions between the host’s health and the hemolymph microbial community, highlighting the factors that may drive the microbiota’s persistence and the host’s physiological homeostasis. Additionally, we outline the cutting-edge techniques currently used to study these microbiomes, addressing the scientific challenges and methodological constraints inherent in hemolymph microbiome research. We aim to present a comprehensive view of current knowledge of the intricate web of interactions within the hemolymph microbiome to elucidate its potential roles in shaping the host’s physiological processes, immune function, and ecological adaptability.

## Hemolymph microorganisms in aquatic invertebrates

The hemolymph microbiome in crustaceans and mollusks exhibits distinct diversity and varying abundance and composition across species and between individuals. This diversity encompasses a wide variety of microbial taxa, which can be categorized as beneficial, commensal, or occasionally even pathogenic [24, 33–35]. Moreover, certain microbial groups are common, whereas others are detected only in specific host species or individuals, potentially reflecting ecological adaptations and immune interactions unique to each organism. However, as mentioned above, earlier studies that relied on culture-dependent methods likely underestimated the “true” diversity due to the nonculturability of many bacteria [26]. Consequently, culture-independent techniques, such as 16S rRNA gene amplicon sequencing, have addressed these limitations and now provide a more comprehensive view of these microbial communities, much more effectively capturing the major microbial groups present in the hemolymph of aquatic invertebrates [26, 31]. Whereas both culture-independent and culture-dependent techniques exhibit inherent biases and limitations, we summarize data from both approaches here to achieve a more integrative understanding of the major bacterial taxa within the hemolymph microbiomes of aquatic invertebrates.

### Crustaceans

The hemolymph of marine crabs hosts a diverse range of microbial communities, predominantly represented by the phyla *Bacteroidota*, *Pseudomonadota*, *Bacillota*, and *Planctomycetota*, as observed in species such as *Scylla paramamosain*, *Eriocheir sinensis*, and *Callinectes sapidus* [27, 36–40]. Similarly, in other commercially important crabs, including *Portunus pelagicus*, *Charybdis feriatus*, *P. sanguinolentus*, and *C. lucifera*, the hemolymph was also found to be dominated by the phyla *Pseudomonadota* and *Bacillota* [41]. The hemolymph microbiota of Pacific white shrimp (*Litopenaeus vannamei*) is also dominated by similar microbial phyla [26, 42]. Furthermore, at the genus level, the distribution across these crustaceans reveals a consistent prevalence of *Pseudomonas*, *Acinetobacter*, *Vibrio*, *Pseudoalteromonas*, *Aeromonas*, and *Arcobacter* [26, 27, 36–40].

### Mollusks

In the oysters, *Crassostrea gigas*, *C. angulata*, and *Saccostrea glomerata*, the hemolymph microbiota is primarily composed of bacterial taxa belonging to the phyla *Pseudomonadota*, *Bacteroidota*, and *Bacillota* [26, 33, 35, 43]. The bacterial genera *Vibrio*, *Pseudomonas*, *Shewanella*, and *Photobacterium* are consistently identified as predominant members of the microbiota in these oysters. Similarly, the hemolymph microbiota of scallops, including *Argopecten purpuratus* and *Patinopecten yessoensis*, is dominated by bacterial taxa from the phyla *Pseudomonadota*, *Bacteroidota*, and *Spirochaetota* [19, 44, 45]. Moreover, along with *Vibrio* and *Pseudomonas*, the genus *Pseudoalteromonas* has also been identified in the hemolymph microbiota of these scallops [19, 44, 45]. Likewise, in mussels such as *Mytilus galloprovincialis* and *M. coruscus*, the hemolymph microbiota is commonly dominated by the phyla *Bacteroidota*, *Pseudomonadota*, *Bacillota*, *Actinomycetota*, and *Verrucomicrobiota* [46–48]. Similarly to scallops, the hemolymph microbiota of mussels is also dominated by the bacterial genera *Vibrio*, *Pseudomonas*, and *Pseudoalteromonas* [47, 48].

### Source of hemolymph microbiota

In addition to delivering nutrients and removing waste, hemolymph also serves as a pathway for bacterial entry and proliferation within the organism. Filter-feeding invertebrates, such as mussels and oysters, obtain nutrients by feeding on smaller organisms suspended in the water, including bacteria, microalgae, and zooplankton, some of which may interact with or influence the hemolymph microbiota. Similarly, it has been reported that the microorganisms present in the surrounding environment primarily shape the structure of host-associated microbial communities [49]. Some of these bacteria may translocate into the hemolymph from the digestive system [27].

The exoskeleton and surrounding tissues serve as the first line of defense against pathogens. Physical injuries can breach these barriers, whether from predation, environmental stressors, or other factors, allowing bacteria direct access to the hemolymph [50, 51]. It has been reported that the hemolymph of healthy aquatic invertebrates typically contains a diverse microbiome that can help fend off infections. However, when the host organisms experience stress or injury, the microbial community can shift drastically, often resulting in lower diversity and increased dominance of pathogenic species in the hemolymph [35, 52]. For instance, infections with entomopathogenic fungi *Metarhizium rileyi* in *Helicoverpa armigera* larvae induce gut bacteria translocation into the hemocoel, which may, depending on the translocated organism, lead to enhanced antibacterial activity and potentially disease [53].

Some bacteria may be transmitted vertically from parent to offspring, establishing a microbiome that includes specific bacterial taxa in the hemolymph from early developmental stages. Early colonization is vital for establishing a stable microbiome that can influence the development of the host’s immune system and overall health [7, 54]. Certain bacteria possess adaptations that evade the host’s immune defenses. This includes mechanisms such as protease secretion that degrade host antimicrobial peptides or altering surface properties to reduce recognition by immune cells [55]. Indigenous bacteria present in other parts of the body (e.g. gut, gill) may also enter and persist in the hemolymph as part of a symbiotic relationship, contributing to the host’s health and immune function despite coexisting with potential pathogens. Hence, the entry of microorganisms into the hemolymph of aquatic invertebrates represents a complex process shaped by environmental factors, physiological vulnerabilities, and interactions with beneficial and pathogenic microorganisms. A deeper understanding of the fate of hemolymph microorganisms is crucial for assessing the dynamics of host health and disease in aquatic ecosystems.

**Figure 1:**
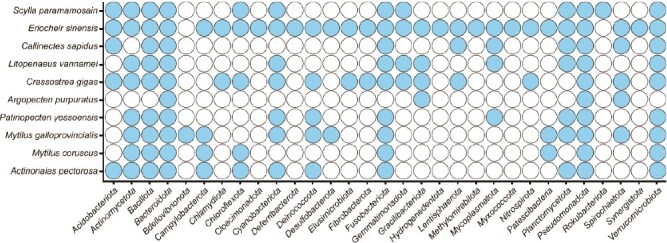
Distribution of bacterial phyla in hemolymph microbiomes of crustaceans and mollusks. Rows represent bacterial phyla identified in the hemolymph of crustacean and mollusk species. Filled circles indicate that members of the corresponding microbial phylum were detected, whereas empty circles indicate that members of this phylum were not detected. The pattern reflects both shared and host-specific bacterial groups shaped by environmental conditions, host immune responses, and long-term host–microbe interactions. Host species included in this comparison are: *Scylla paramamosain* [37, 38], *Eriocheir sinensis* [40], *Callinectes sapidus* [36], *Litopenaeus vannamei* [42], *Crassostrea gigas* [33, 35], *Argopecten purpuratus* [19], *Patinopecten yessoensis* [44], *Mytilus galloprovincialis* [57], *M. coruscus* [48], and *Actinonaias pectorosa* [46]. All data were obtained using culture-independent methods. Specifically, 16S rRNA gene amplicon sequencing was used in all studies except *C. sapidus* [36], which used a 16S rRNA gene clone library. The figure was generated using SRplot (https://www.bioinformatics.com.cn/en).

Overall, the hemolymph microbiomes of crustaceans and mollusks are distinct from those of other body sites and the surrounding environment [40, 56]. Though diversity and composition can vary among species, these communities often share key taxonomic groups, suggesting the influence of common ecological functions and evolutionary pressures (Fig. 1). It is important to remember that aquatic invertebrates can have a very rich microbial diversity (≳300 genera) compared to terrestrial invertebrates such as insects (≳45 genera), primarily attributed to their extensive microbial exposure in aquatic habitats [58, 59]. Water acts as a dynamic medium, facilitating the constant exchange of microorganisms between the host and its environment. Aquatic invertebrates are frequently in contact with diverse microbial communities present in water columns, sediments, and biofilms. These environments harbor many bacteria, fungi, and other microbes, which can colonize the hemolymph either passively through physical interactions or as part of intimate host–microbe associations. In addition to microbial composition, environmental parameters such as temperature and pH can significantly influence microbial community dynamics in the surrounding water, thereby shaping the structure and diversity of the host’s hemolymph microbiome [34, 48, 60]. However, the consistent and widespread exposure to a microbially dense environment may significantly contribute to the diversity of their hemolymph microbiome. As outlined above, hemolymph microbiomes of aquatic invertebrates are composed of various bacterial phyla, including *Pseudomonadota*, *Bacteroidota*, *Bacillota*, *Spirochaetota*, *Verrucomicrobiota,* and *Actinomycetota*, which are prevalent across various species. However, as mentioned in the Crustaceans and Mollusks sections, one of the most prominent features of these microbiomes is the consistent presence of potentially pathogenic species, including those from the genera *Vibrio*, *Pseudomonas*, *Aeromonas*, and *Shewanella*, which are commonly detected in the hemolymph of both crustaceans and mollusks. These genera contain previously reported opportunistic pathogens that can cause infections in stressed or immunocompromised hosts and contribute significantly to marine diseases. Some *Vibrio* species, such as *V. anguillarum*, *V. alginolyticus*, *V. cyclitrophicus*, and *V. parahaemolyticus*, can be pathogenic to marine organisms, causing disease through direct environmental exposure, often via waterborne transmission or colonization of external tissues. In addition, species like *V. parahaemolyticus* and *V. alginolyticus* are also implicated in human infections, which are primarily acquired through the consumption of infected or undercooked seafood [61]. Moreover, some other opportunistic pathogens, including members of the *Vibrio* and *Photobacterium* genera, became relatively more abundant, especially during environmental shifts such as changes in salinity [62]. Similarly, *Aeromonas* species such as *A. hydrophila*, *A. caviae*, and *A. veronii* have been frequently associated with disease outbreaks in marine and aquaculture organisms, including mussels, oysters, and shrimp, where they adversely affect host health and immune responses through virulence factors and antibiotic resistance traits [63, 64]. The ubiquity of these potentially pathogenic strains across a wide range of host species emphasizes the importance of microbial balance in maintaining host health, as disruptions to this balance can lead to pathogen proliferation. Furthermore, the widespread presence of these pathogens suggests that the hemolymph microbiome is not only critical for ecological and immune interactions but also a key player in the susceptibility of these organisms to disease outbreaks, indicating the need for further studies to understand the role of these microbial communities in health and disease dynamics.

## Factors influencing the diversity of hemolymph microorganisms

**Figure 2:**
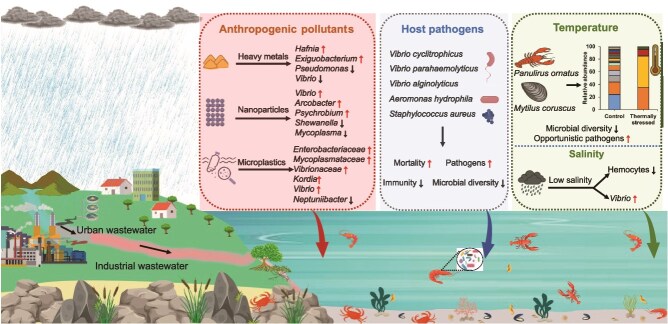
The schematic diagram illustrates the various stressors influencing the hemolymph microbiome of aquatic invertebrates, including anthropogenic compounds, environmental changes, and host-associated pathogens. The illustration was created with selected graphical elements from BioRender (https://www.biorender.com).

Various biotic and abiotic factors are significantly linked to the microbiome’s structure, function, and composition. Elucidating the mechanistic basis for these links is a crucial challenge for developing a thorough understanding of microbial community dynamics in the hemolymph in the future. The microbial community composition is dynamic, with short-term responses to abiotic and biotic factors and long-term stability influenced by microbiome interactions and host population–specific factors [35]. Surrounding environments play a critical role; changes in ambient water temperature can affect the metabolic rates of both invertebrates and their microbial symbionts, leading to community shifts that favor temperature-resilient microbes [33]. In addition, environmental pollutants such as heavy metals, pesticides, nanoparticles, and other industrial chemicals; the emergence of pathogens; ocean acidification; and changes in salinity and seasons can all have a significant impact on microbial survival and growth, which will subsequently influence the microbial community structure in hemolymph (Fig. 2).

### Effect of environmental conditions (temperature, salinity, pCO_2_)

The diversity of the hemolymph microbiota seems strongly influenced by environmental factors, although the microbial communities differ significantly between hemolymph and seawater [33]. Environmental factors such as temperature change significantly drive the bacterial community structure and dynamics in the hemolymph of aquatic invertebrates. It was recently reported that an increase in temperature from 28°C to 34°C does not majorly affect the survival or immune parameters of the lobster (*Panulirus ornatus*); however, significant differences in the hemolymph microbiome composition were observed. For example, the relative abundances of *Loktanella*, *Cohaesibacter*, *Polaribacter*, and *Micrococcus* were higher in the control group, whereas the thermally stressed group showed a significant decrease in both abundance and diversity across all taxonomic levels [34]. Similarly, an increase in experimental temperature from 21°C to 27°C significantly reduced the microbial diversity in the hemolymph of *M. coruscus*, as indicated by decreases in Chao1, Shannon, and Simpson indices at 27°C. At this elevated temperature, the microbial composition also shifted, with increased relative abundance of genera such as *Vibrio* and *Amphritea*, and decreased abundance of *Bacillus* and *Pseudomonas* [48]. Moreover, elevated pCO_2_ levels significantly increased the diversity and richness of the hemolymph microbiome of *S. glomerata* oysters, whereas temperature fluctuation only increased species richness, indicating that these stressors may have individual rather than combined impacts on oyster hemolymph microbiomes [43]. In addition to these experimental approaches, wild-collected samples support the theory that environmental conditions shape the abundance and composition of hemolymph microbiomes. Recently, it was observed that the abundance of marine heterotrophic bacteria in oyster hemolymph increases during spring and summer compared to other seasons [65]. Similarly, seasonal changes significantly influenced the hemolymph microbiota composition and immune parameters in *Mytilus galloprovincialis*, with water temperature identified as a crucial factor in shaping microbial communities, particularly affecting specific bacterial genera [47]. Environmental stressors, such as elevated temperature and decreased salinity, altered the hemolymph microbial community of *C. virginica*; *V. aestuarianus* dominated in the low-salinity/high-temperature group, whereas *V. parahaemolyticus* disappeared by the end of the 28^th^ day exposure period, indicating dynamic microbial shifts [60]. The abundance of hemocytes in *P. homarus* dropped significantly at a salinity of 15 compared to 35, indicating potential immune system impairment [62]. Additionally, no significant differences were observed in the total heterotrophic bacterial count in the hemolymph of *Pinctada radiata* across salinity levels, whereas *Vibrio* spp. counts were significantly higher in the low salinity, indicating that lower salinity may alter the microbial community and affect the oyster’s health and immune response [66].

### Influence of pathogens

Pathogenic organisms in the hemolymph indicate host stress or disease, which is generally caused by poor immune regulation [25]. Under normal conditions, the immune system maintains microbial homeostasis by suppressing opportunistic pathogens. However, as the disease progresses, immune regulation weakens, resulting in microbial imbalances and the proliferation of opportunistic taxa in the hemolymph microbiome. These disruptions jeopardize microbial stability, exacerbate physiological stress, and impair immune function, indicating the critical link between host health and hemolymph microbiome composition. For instance, exposure to waterborne *V. cyclitrophicus* was found to cause high mortality in mussels *M. coruscus* with simultaneous reduction in the microbial diversity of their hemolymph [48]. Similarly, it was observed that the *Vibrio* infection led to an increase in the abundance of *Gammaproteobacteria* within scallop hemolymph, whereas *Epsilonproteobacteria*, often associated with a healthy microbiome, were decreased [19]. Moreover, distinct microbial community shifts were observed in each *A. hydrophila* and *Staphylococcus aureus* pathogenic infection in the hemolymph of Chinese mitten crab [39]. In addition, it was observed that mud crabs (*S. paramamosain*) with whitish muscle syndrome exhibit higher hemolymph bacterial diversity than healthy ones, with no significant difference in fungal communities [67].

### Effect of anthropogenic compounds

Chemically synthesized industrial compounds, often categorized as xenobiotics due to their toxicity to living organisms, primarily originate from anthropogenic activities such as industrialization, agriculture, and urban development [68]. However, natural processes such as volcanic eruptions and forest fires also release xenobiotics into the environment [69]. These compounds include a wide range of pollutants such as engineered nanoparticles, microplastics, heavy metals, and various structurally different chemicals designed for specific applications with long half-life and low biodegradability [70–72]. Due to their persistence, these xenobiotics accumulate in the environment and enter aquatic ecosystems through agricultural runoff, improper industrial wastewater discharge, and atmospheric deposition [73, 74]. Once they enter aquatic habitats, these compounds disrupt ecosystems by altering microbial communities essential for host health, nutrient cycling, and ecosystem functioning. Moreover, by processing large volumes of water, filter-feeders can concentrate xenobiotics such as heavy metals, microplastics, and other pollutants within their tissues, making pollutant exposure particularly impactful on their ecology [75, 76].

Exposure to TiO_2_ nanoparticles significantly altered the hemolymph microbial community of *M. galloprovincialis*, with a control group exhibiting a more diverse core microbiome compared to a nanoparticle-exposed group with specific genera showing substantial changes in relative abundance following exposure [77]. Similarly, exposure of *M. galloprovincialis* to aminomodified nanopolystyrene led to increased relative abundance of potentially pathogenic genera such as *Arcobacter*, *Psychrobium*, and *Vibrio*, accompanied by decreases in the relative abundance of previously dominant genera like *Shewanella* and *Mycoplasma*. Although the latter taxa can include opportunistic pathogens, their relative decline, together with immune suppression, suggests a nanoplastic-induced shift in the hemolymph microbiota that may favor pathogenic over commensal or resident communities, potentially disrupting host–microbiota homeostasis [78]. Additionally, significant changes were observed in the hemolymph microbiota of *Artemia* after exposure to amino-modified polystyrene nanoplastics, with *Neptuniibacter* (21.33%) and *Vibrio* (14.48%) dominant in the control group, whereas acute exposure shifted dominance to *Kordia* (28.38%) and *Vibrio* (19.84%). Chronic exposure maintained *Kordia* (10.78%) and *Vibrio* (15.15%), indicating that it has enormous potential to disrupt the natural microbiota balance [79]. Compounds naturally found in some environments, such as hydrogen sulfide, can also act as pollutants. For example, the hemolymph microbiota of *Tegillarca granosa* was found to include diverse bacteria, primarily composed of *Pseudomonadota*, *Spirochaetota*, *Bacteroidota,* and *Bacillota,* similar to other bivalves. Experimental sulfide stress altered this microbiota composition, enabling the growth of opportunistic pathogens such as *Vibrio* and *Pseudoalteromonas*, whereas the beneficial members, such as *Lactobacillus* and *Desulfovibrio*, were reduced [80]. Microplastics, a xenobiotic composed of synthetic polymers, have become widespread environmental contaminants and are of concern due to their persistence, small size, and ability to accumulate in ecosystems, which pose significant risks to aquatic organisms and food webs. For instance, exposure to polyethylene microplastics led to an increased abundance of specific bacterial families such as *Enterobacteriaceae*, *Mycoplasmataceae*, and *Vibrionaceae* in crayfish, indicating a potential impact of microplastic exposure on its hemolymph microbiome [81]. Similarly, polyester microfibers were observed to significantly affect the hemolymph microbiome of *M. galloprovincialis*, leading to a decrease in *Pseudomonadota* and *Campylobacterota*, with an increase in *Bacteroidota* and a reduction in the *Bacillota*/*Bacteroidota* (F/B) ratio. These changes are more pronounced at higher concentrations (100 μg/l) [57]. Significant changes in hemolymph bacterial composition were observed in *Procambarus clarkii* after exposure to hexavalent chromium [Cr (VI)], with increased abundance of *Hafnia* and *Exiguobacterium* and decreased levels of *Pseudomonas* and *Vibrio* [82]. The hemolymph microbiome of *L. vannamei* shrimp was primarily composed of *Pseudomonadota*, *Bacteroidota*, and *Bacillota*, with *Vibrio* as the dominant genus. Although the composition varied with different water ammonia nitrogen (ammonia-N) concentrations, no significant differences in species abundance were observed; however, the prevalence of genera like *Achromobacter* and *Burkholderia* suggests potential health risks to shrimp from prolonged ammonia-N exposure [83].

## Immune response and established defense mechanisms

Over the last decade, the high demand for various crustacean and mollusk species for human consumption has driven extensive research into their immunodynamics. Understanding the immunity of these animals is crucial for sustainable farming and providing for human nutritional needs. The innate immune system of those aquatic invertebrates that have been studied is considered robust and exceptionally evolved to respond to pathogens and xenobiotics quickly, ensuring cellular homeostasis despite the absence of the adaptive immune system found in vertebrates [84–87]. Although considered a core component of the immune system, hemocytes found in invertebrate hemolymph (primarily granulocytes and hyalinocytes) play an important role in a variety of host physiological functions, including nutrient transport, breathing, excretion, tissue repair, and immune defense [86, 88, 89]. Antimicrobial peptides (AMPs) are key effector molecules of the invertebrate immune response, offering broad-spectrum protection against bacterial, viral, and fungal pathogens. These peptides are rapidly produced and secreted by hemocytes upon immune challenge. Research across various crustacean and mollusk species has revealed a wide diversity of AMP families and structures, indicating a conserved yet adaptable antimicrobial strategy [90–106]. AMPs act directly by disrupting pathogen membranes and function in immune signaling and modulation.

Recent findings have expanded our understanding of hemolymph immunity by highlighting the role of resident microbial communities. These symbiotic or commensal microbes can enhance host immune defense by producing bioactive compounds such as cyclolipopeptides and nonribosomal peptides with antimicrobial activity [107, 108]. This synergy between host cells and microbiota contributes to the overall stability and effectiveness of the immune system. Several intracellular signaling pathways and transcription factors are crucial to regulating AMP expression and other immune responses. These include the signal transducers and activators of the transcription (STAT) pathway [109], mitogen-activated protein kinase (MAPK) cascades [110, 111], and transcription factors such as forkhead box O (FOXO) [112] and Krüppel homolog 1 (Kr-h1) [113]. Upon pathogen exposure, these pathways become activated to induce immune gene transcription, enabling a swift and specific response to microbial threats. In addition to effector mechanisms, invertebrates possess a range of pattern recognition receptors (PRRs) that identify conserved microbial structures known as pathogen-associated molecular patterns (PAMPs). These receptors, including Toll-like receptors, lectins, lipopolysaccharide-binding proteins (LPSs), and thioester-containing proteins (TEPs), initiate immune cascades leading to phagocytosis, AMP production, and inflammatory signaling [87, 114–116]. Moreover, invertebrate immune function is not static. It responds dynamically to factors such as environmental stress and molting cycles (if present), which can modulate immune gene expression and cellular activity [111, 117–121]. A detailed overview of immune components, regulatory mechanisms, and associated species is provided in Table 1. These findings underscore the complexity, adaptability, and ecological relevance of the hemolymph immune system in aquatic invertebrates, forming a strong foundation for applications in aquaculture health and disease management.

**Table 1:** Characterized hemolymph-derived immune molecules, signaling pathways, and regulatory mechanisms in crustaceans and mollusks.

**Species**	**Immune component**	**Function and mechanistic insight**	**Reference**
*Mytilus coruscus* (Thick shell mussel)	Myticofensins	Significant upregulation of multiple genes from this AMP family was observed in hemocytes following bacterial and fungal infection, indicating their role as major AMP-producing cells	[90]
*Fenneropenaeus indicus* (Indian white prawn)	*Fi*-crustin2	A cationic AMP from hemocytes with potent antibacterial activity against Gram-negative bacteria	[92]
*Scylla serrata* (Mud crab)	*Ss*-arasin	A hemocyte-derived AMP exhibiting broad-spectrum antimicrobial activity against Gram-positive and Gram-negative bacteria	[97]
*Scylla paramamosain* (Green mud crab)	Crustins (*Sp*Crus2, *Sp*Crus3–5, *Sp*Crus8), *Sp*PR-AMP1, SCY2, SCY4, SCY5, *Sp*Hyastatin, *Sp*gly-AMP, Scyreprocin, Scyreptin 1–30, Spasin 141–165	Diverse AMPs expressed predominantly in hemocytes; exhibit potent antimicrobial activity against Gram-positive and Gram-negative bacteria and function in both localized and systemic immune defense	[93–96, 98–105]
*Dromia dehaani* (Sponge crab)	Dromidin	A hemolymph-derived AMP with broad-spectrum antimicrobial activity against bacterial and fungal pathogens	[122]
*Crassostrea gigas* (Pacific oyster)	Alterins (produced by hemolymph-associated *Pseudoalteromonas* strains)	Cyclolipopeptides binding to LPS, causing membrane depolarization and bacterial lysis; active against Gram-negative pathogens	[107]
*Hyriopsis cumingii* (Triangle sail mussel)	*Hc*STATs (STAT1, STAT2, and STAT3)	Bacterial challenge triggers STAT pathway activation in hemocytes, regulating AMP gene expression and enhancing immune responses	[109]
*Eriocheir sinensis* (Chinese mitten crab)	*Es*MEK	Bacterial challenge activates the MEK–ERK pathway in hemocytes, regulating AMP gene expression and enhancing immune responses	[110]
*Marsupenaeus japonicus* (Kuruma shrimp)	FOXO	Induced upon *Vibrio anguillarum* infection; drives AMP gene expression and enhances hemocyte phagocytosis, contributing to antibacterial immunity	[112]
*Penaeus vannamei* (White shrimp)	*Pv*Kr-h1	Regulates oxidative stress and AMP gene expression by enhancing ROS production and promoting Relish activation during bacterial infection	[113]
*Chlamys farreri* (Zhikong scallop)	*Cf*LRRop-7 (LRR-only PRR)	Recognizes PAMPs, promotes lysozyme activity, and regulates AMP gene expression, contributing to antibacterial immunity	[115]
*S. paramamosain*	*Sp*-AST-B (B-type allatostatin neuropeptide)	Enhances innate immunity by promoting nitric oxide production, phagocytosis, and immune gene expression in response to bacterial and viral challenges	[117]
*S. paramamosain*	*Sp*p38 (p38 mitogen-activated protein kinase)	Regulates ALF expression and ROS production, promoting antibacterial immunity, and maintaining hemolymph microbiota homeostasis during bacterial infection	[118]
*Portunus trituberculatus* (Swimming crab)	Signal transduction molecules (MAPK cascade, Toll pathway components), PRRs, and antioxidative enzymes	Differential expression across molting stages; regulate pathogen recognition, immune signaling, and oxidative stress defense during molting	[111]
*Macrobrachium rosenbergii* (Giant river prawn)	C-type lectins (*CLEC1*, *LEC3*), *ALF2*, Cu/Zn *SOD*, *HSP70*, and *hemocyanin1*	Upregulated during *Enterobacter cloacae* infection; involved in pathogen recognition, oxidative stress response, and immune regulation through phagosome and lysosome pathways	[119]
*Argopecten purpuratus* (Chilean scallop)	*Ap*Glys	G-type lysozyme hydrolyzes bacterial cell walls, promotes antibacterial defense, and maintains microbial homeostasis in scallop hemolymph	[45]
*S. paramamosain*	*Sp*-Spz	Spätzle gene is a key regulator in the Toll pathway that promotes ALF expression, supports hemolymph microbiota homeostasis, and maintains hepatopancreatic tissue integrity during immune responses	[120]

ALF, anti-lipopolysaccharide factor; AMPs, antimicrobial peptides; ERK, extracellular signal-regulated kinase; FOXO, Forkhead box O; LPS, lipopolysaccharide-binding proteins; MAPK, mitogen-activated protein kinase; MEK, mitogen-activated protein kinase kinase; PAMPs, pathogen-associated molecular patterns; PRRs, pattern recognition receptors; ROS, reactive oxygen species; STAT, signal transducers and activators of the transcription.

## Immune evasion and survival of microorganisms in hemolymph

**Figure 3:**
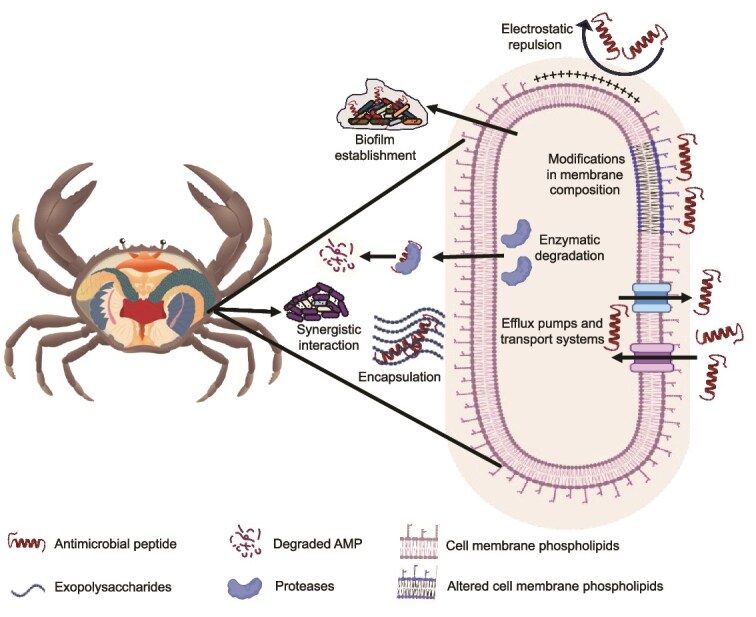
Schematic representation of the mechanisms of antimicrobial peptide (AMP) resistance in hemolymph-associated bacteria: bacteria evade AMPs through multiple strategies, including modifications in membrane composition, active efflux pumps, enzymatic degradation of AMPs, biofilm formation, encapsulation, and synergistic interactions with the host microbiota. The figure was prepared using a crab anatomical illustration obtained from Adobe Stock (https://stock.adobe.com) and graphical components from BioRender (https://www.biorender.com).

As discussed in the previous sections, a taxonomically diverse microbial community persists in the hemolymph of crustaceans and mollusks despite the presence of broad-spectrum AMPs and hemocyte-mediated immune responses like phagocytosis and encapsulation [123, 124]. This persistence suggests that microorganisms have evolved a variety of sophisticated mechanisms and strategies to evade host immune defenses successfully (Fig. 3). For instance, the most common mechanism of AMP resistance is altering the cell membrane structure by incorporating positively charged molecules, which decreases the binding and interaction of cationic AMPs [125]. Similarly, the proteolytic degradation of AMPs by extracellular proteases, such as protease IV, alkaline protease, elastase A, and elastase B, represents a simple yet effective mechanism for providing AMP resistance to microorganisms, thereby playing a predominant role in promoting bacterial development within the infected host [126]. Moreover, by modifying the molecular structure of their PAMPs, such as LPS, bacterial pathogens can evade detection by the host’s PRRs and also reduce the activation of downstream immune signaling pathways [127]. Similarly, some pathogens employ molecular mimicry by expressing surface molecules that resemble host proteins, allowing them to evade immune recognition and impair the activation of immune responses [128, 129]. In addition, bacteria utilize efflux pumps to expel AMPs from their cytoplasmic membrane, reducing their disruptive effects and increasing resistance to host immune defenses [130]. Additionally, biofilm formation within the hemolymph provides pathogens with a protective niche, where the extracellular polymeric matrix (composed of proteins, DNA, and exopolysaccharides) limits AMP penetration and enhances microbial resistance and persistence [131, 132]. Moreover, some microorganisms within the hemolymph establish symbiotic relationships with the host that minimize immune activation, often producing antimicrobial compounds that protect the host from pathogenic microbes. Nevertheless, as mentioned in the Immune Response and Established Defense Mechanisms section, although many microbes possess strategies to evade immune detection, these mechanisms are often incomplete or transient. The host immune system can still detect microbial presence through a diverse array of PRRs that recognize conserved PAMPs or damage-associated molecular patterns (DAMPs). This recognition activates intracellular pathways such as MAPK and STAT, resulting in the upregulation of AMPs and other immune effectors. Therefore, even when facing immune-evasive microbes, the hemolymph maintains an active immune surveillance system capable of initiating effective defense responses.

## Multi-omics reveal complicated interplay between host and microbiota

**Figure 4:**
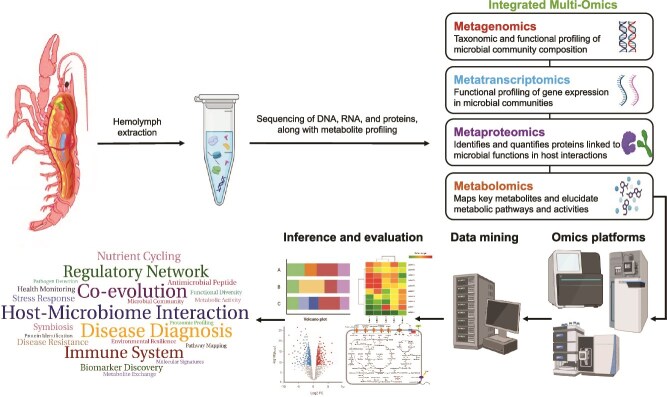
Integrated omics strategies provide a comprehensive framework for deciphering the hemolymph microbiome and its functional dynamics in aquatic invertebrates: the schematic diagram demonstrates the merits of employing an integrated multi-omics approach to unravel the host–microbe interactions, regulatory network, and various other functions of hemolymph microbiome in aquatic invertebrates. The illustration was prepared using a lobster image from Shutterstock (https://www.shutterstock.com) and additional graphical components from BioRender (https://www.biorender.com).

A range of techniques has been used to study the hemolymph microbiome. Initially, culture-based techniques were predominant; however, these methods have well-characterized limitations in accurately representing microbial abundance and diversity [26]. Therefore, culture-independent approaches such as metagenomics, metatranscriptomics, metaproteomics, and metabolomics have been increasingly applied to analyze various microbial ecosystems over the past decade. These methods have revolutionized the field of microbiome research by providing a more comprehensive understanding of microbial diversity, functional potential, and activity. An in-depth understanding of the hemolymph microbiome can only be achieved through integrating these individual omics approaches into a unified multi-omics framework (Fig. 4). For example, a major disease affecting Akoya oysters was investigated through metagenomic analysis, using 16S rRNA gene and shotgun sequencing, which ultimately revealed genera within the *Spirochaetes* phylum as potential pathogens [31].

Proteomic analysis of *E. sinensis* hemolymph after *Metschnikowia bicuspidata* yeast infection showed the immune activation and potential suppression of regeneration, elucidating crustacean molecular responses to fungal pathogens [133]. Hemolymph metabolomic analysis of marine blue swimmer crab (*P. pelagicus*) and mud crab (*S. tranquebarica*) showed a rich diversity of bioactive compounds, including tannins, sappanones, and upregulated proteins, indicating a solid host defense mechanism against pathogens [134]. Moreover, distinct metabolite profile differences were observed in the hemolymph of *Vibrio*-infected and -uninfected mussels, as well as between genders, with seven key metabolites, including itaconic acid, isoleucine, and phenylalanine, differing significantly in female mussels recovering from infection [28]. Similarly, in another study, postharvest handling of greenshell mussels was found to elevate stress-related metabolites, particularly those involved in energy production, with a recovery process initiated after a three hour transport period and further enhanced by subsequent re-immersion [135]. Significant changes in amino acid biosynthesis and metabolism were observed in marine mussel hemolymph exposed to ocean acidification, indicating susceptibility to environmental stress and emphasizing the importance of metabolomics for understanding molecular mechanisms and supporting environmental risk assessment and sustainable aquaculture strategies [136]. These examples show the depth of understanding that can be achieved with omics analyses; however, they lack the integrative depth needed to fully resolve the complex host–hemolymph microbiome interactions.

Integrating several omics techniques has the potential to provide an even more comprehensive understanding, particularly of host–microbe interactions, because such interactions inherently involve multiple species. For example, combined transcriptomic and metabolomic analysis of *V. parahaemolyticus* infected *S. paramamosain* mud crabs showed significant changes in gene expression and metabolic pathways such as amino acid metabolism, aminoacyl-transfer ribonucleic acids (aa-tRNAs) biosynthesis, and the tricarboxylic acid (TCA) cycle, thereby underscoring the complex adaptations of mud crabs to pathogen exposure and demonstrating the value of “omics” methods in decoding marine crustacean host–pathogen interactions and disease resistance [137]. Similarly, significant changes in the transcriptome and metabolome of female swimming crabs exposed to high ammonia were identified, with 589 genes and 72 metabolite alterations, emphasizing the crab’s adaptation to environmental stress and showing the impact on immune and stress responses, energy metabolism, and cellular stability [138]. Moreover, transcriptome and metabolome analyses of *E. sinensis* during different molting stages showed that crabs with a higher weight gain rate had more active amino acid, nucleotide, and energy metabolism [139]. This group also displayed enhanced protein biosynthesis, neuroendocrine activity, and antioxidant capabilities. These traits are linked to increased weight gain, providing insights into growth mechanisms relevant to aquaculture and breeding strategies. In addition, transcriptome and metabolome analyses in juvenile mud crabs during molting revealed disruptions in hormone and immune functions due to *S. aureus* and *V. alginolyticus* infections. These disruptions lower cortisol and activate immune responses with significant upregulation of genes like C-type lectin, scavenger receptor, fibronectin, and i-type lysozyme, showing how bacterial infections affect molting in crabs and exhibit the critical interaction between immune genes and hormones under bacterial stress [140]. Multi-omics studies not only report parallel shifts in gene expression and metabolites but also reveal correlations between specific immune or hormone-related genes and metabolic alterations. For example, pathway-level integration and correlation analyses in these datasets have linked immune responses to shifts in amino acid biosynthesis, neuroendocrine signals, and growth performance, demonstrating how integrated multi-omics approaches can uncover functional relationships between microbial stress, host immune signaling, and physiological adaptations [137–140].

## Future prospective

The hemolymph microbiota of crustaceans and mollusks is increasingly recognized for its essential role in host immune functions, influencing their overall health and resilience. However, current studies have focused solely on bacteria, leaving a gap in knowledge about the potential roles of archaea and fungi in the hemolymph microbiome. Exploring whether archaeal and fungal species are common components of the hemolymph microbiome and understanding their potential contributions to immune homeostasis may reveal new aspects of host–microbe interactions. Similarly, elucidating the presence of a core, resilient hemolymph microbiome in aquatic invertebrates under homeostatic conditions is critical for understanding fundamental immunological and physiological states in these organisms. Conditions that promote either the stabilization or dysbiosis of the hemolymph microbiome can be identified by investigating how diet, stocking density, and aquaculture related stressors (e.g. immunological challenges and environmental perturbations) modulate microbial community composition. Techniques that go beyond relative abundance will be crucial for such efforts. Moreover, the dynamic nature of microbial communities suggests that hemolymph microbiota may vary throughout the developmental stages of the host. Therefore, future studies should focus on longitudinal sampling across life stages to identify age- or stage-specific microbiome compositions, such as potential life-stage discriminatory lineages in the hemolymph. This will enable focused interventions at critical life stages, particularly in aquaculture, where early-stage health is crucial.

The utilization of beneficial microorganisms within the hemolymph as microbial agents for disease prevention in aquaculture represents a paradigm shift in disease management, with the potential to reduce antibiotic dependency, mitigate economic losses from pathogen outbreaks, and improve the sustainability of aquaculture practices. Moreover, RNA-based biopesticides have also shown great potential in combating disease outbreaks by specifically targeting pathogenic gene expression, offering a sustainable and precise alternative to conventional antimicrobial strategies [141]. However, a thorough understanding of hemolymph microbial communities and their functional roles could facilitate the development of targeted probiotic or microbiome-modulating agents designed to improve immune function, reduce pathogen burden, and mitigate pathogen-associated economic losses in aquaculture. In parallel, future efforts should use microbiota data from various species to predict beneficial bacteria and introduce them during larval rearing to enhance the synergistic interactions between the hemolymph microbiome and the innate immune system for long-term protection. Targeted therapeutic interventions, including antibiotics, could be optimized based on the specific microbial profiles identified in different invertebrate species.

Using experimental approaches to demonstrate further that the microbes detected in the hemolymph represent a resident, active population is another important focus for future research. Although culture-independent techniques such as metagenomics and metatranscriptomics have consistently revealed stable, host-specific microbial communities in the hemolymph of aquatic invertebrates, these microbes active metabolic and functional roles remain less explored. Evidence of microbial gene expression and metabolite production in hemolymph suggests that these populations are metabolically active and interact with the host immune system, contributing to homeostasis and pathogen defense [19, 142, 143]. However, further studies using, e.g. longitudinal sampling, controlled experiments with axenic invertebrates, fluorescently labeled microorganisms coupled with imaging, and the various techniques available for imaging microbial activity *in situ* will provide much more detailed insights into their location, metabolism, and function within the host [144–147]. Applying methods to quantify microbes in the hemolymph habitat may also reveal patterns not visible with surveys of relative abundance alone [148]. Investigating their persistence mechanisms, functional roles, and potential co-evolution with the host could offer groundbreaking insights into immune–microbiome dynamics and will provide a robust basis for effective probiotic applications and improved aquaculture sustainability.

Deciphering the mechanisms underlying the synergistic relationship between the hemolymph microbiome and the innate immune system, within and across generations, constitutes an essential future research endeavor and is expected to require highly targeted and multidisciplinary efforts. Critical genes, proteins, and pathways that play a crucial role in regulating these interactions must be identified and characterized to enhance the microbiome-immune system synergy and provide a sustainable strategy for aquaculture. Integrating these research avenues will contribute to a better understanding of hemolymph microbiota dynamics, promoting sustainable aquaculture practices that improve animal health, resilience, and productivity, resulting in healthier aquatic invertebrate populations and more biologically informed aquaculture systems. Finally, just as environmental conditions play a significant role in shaping the hemolymph microbiome, these microbiomes could provide a window into the health status of the surrounding environment. Efforts to develop hemolymph biomarkers based on microbial profiles as novel tools for environmental monitoring are in their infancy but are sensitive and accurate tools for assessing ecosystem health, even in highly remote areas [149, 150].

## Conclusion

The hemolymph of aquatic invertebrates, along with their microbiota, form a complex and dynamic ecosystem that is influenced by various environmental factors and the physiological adaptations of the host. However, in many organisms, the relationship between hosts and their microbiota tends to remain relatively stable over time, with a notable link between the host’s evolutionary lineage and its microbial community, a concept known as phylosymbiosis [151]. This link indicates that hosts shape the structure of their microbiota, leading to similarities between host evolutionary relationships and microbial composition. As a result, the host immune system plays a critical role in moderating these microbial populations, employing innate and adaptive responses to maintain a balanced microbiota and defend against pathogenic invasion. Achieving a balance between tolerance of beneficial microorganisms and suppression of pathogens is one of the primary selective pressures that have driven the evolution of immune strategies and mechanisms in these organisms over prolonged evolutionary periods. Widespread application and advancements in multi-omics approaches, such as metagenomics, metatranscriptomics, metaproteomics, and metabolomics, have revolutionized our understanding of the host–microbe interaction and response, showing that environmental variables such as temperature, pollutants, and pathogen exposure significantly alter the microbial communities within the hemolymph. Moreover, these integrated approaches have provided profound insights into the microbial composition, gene activity, protein functions, and metabolic interactions within hemolymph. A comprehensive understanding of these microbial dynamics and their environmental interactions is vital for advancing aquaculture sustainability and enhancing the health and resilience of aquatic invertebrates. Moreover, the intricate web of interactions within the hemolymph microbiome emphasizes the necessity for continued research and technological innovation to support sustainable aquaculture and improve the welfare of these essential species.

## Data Availability

Data sharing not applicable to this article as no datasets were generated in the current review.
